# Strangulated Umbilical Hernia

**Published:** 1885-02

**Authors:** 


### Selections.

### STRANGULATED UMBILICAL HERNIA.

Dr. H. R. Burrell, in the *Boston Medical and Surgical Journal* of October 16, 1884, reports a very interesting case of strangulated umbilical hernia. The patient was a woman aged fifty-two and weighing 230 pounds. When admitted to the Carney Hospital it was found that she had a strangulated umbilical hernia. The tumor had existed four years, and had always been reducible until for about sixty hours before entrance, and was now of a doughy feel, livid in spots, and of tympanitic resonance. The patient was in intense pain, with a temperature of 101° F. and pulse of 150, small and wiry. An attempt was made, under ether, to reduce the tumor by taxis, but as this was without avail, an operation was decided on. An incision was made, three inches in length, over the superior part of the tumor. When the skin was divided, a large serous exudation escaped, showing that sloughing would have followed in a few hours. The constricting band was divided after some difficulty, owing to the tenseness of the tumor, and the hernia liberated. Upon being reduced there was found considerable adherent omentum remaining. This was removed by ligation. The redundant skin was then removed, leaving an abdominal section four to five inches in length. Hemorrhage was not troublesome. Small sponges were packed into the bottom of the wound to prevent the blood getting into the abdominal cavity. The wound was closed by wire sutures, deep and superficial, and dressed with bichloridized gauze. A binder was also adjusted. The patient did well until the third day, when she became very stupid, with flushed face and contracted pupils. Temperature, 100° F.; pulse, 122. Upon removing the dressing, a quantity of offensive, sanious fluid escaped from the wound. All the sutures were then removed excepting a deep wire one, and the wound thoroughly washed out with chlorinated soda, a drainage tube was then inserted into the cavity, and iodoform used as a dressing. The wound was syringed out night and morning with the antiseptic solution. Upon the ninth day a mass of necrosed tissue was removed and the flaps were found to be undermined. The temperature, kept practically normal, and the thorough escape of the discharge prevented any constitutional disturbance.

Upon the twenty-fourth day after the operation, during the dressing, a supposed piece of necrosed tissue was seized in the forceps, when, to the surprise of all, a small piece of sponge the size of a chestnut was removed. This had been packed beneath the flaps at the time of the operation to control hemorrhage and had been overlooked. The convalescence was somewhat delayed, but she was finally discharged, well. The interesting features of this case were the abdominal section, and the disturbance caused by leaving a small sponge in the wound.

By the abdominal section a penetrating wound was changed into an incised one in which the edges could be brought accurately together, and, as the result showed, perfect union occurred, notwithstanding the aggravating delay. The lesson to be learned from not removing all the sponges from the wound is obvious and need not be enlarged upon. It is hardly possible to be over-cautious in this matter, and in all surgical operations accidents of this kind should be carefully guarded against.

